# 

**Published:** 1881-06

**Authors:** 


A MONTHLY JOURNAL,

DEVOTED TO

### Medical, Surgical, Obstetrical, Dental, Hygienical and Popular Sciences.

S.G.O.L. INDEXED

EDITED BY

HARVEY L. BYRD, A. M., M. D.,

AND

BASIL M. WILKERSON, D. D. S., M. D.

**"Altera poscit opem res, et conjurat amice."**

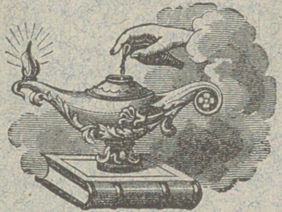

NEW YORK:

*Published by B. M. WILKERSON, Proprietor,*

Office, 20 Courtlandt Street.

Wm. Miller, Business Manager.

**$3.00 Per Annum in Advance. - - Single Copies 30 Cents.**

ENTERED AT THE POST OFFICE, NEW YORK CITY, AS SECOND-CLASS MATTER.

